# Accelerator Mass Spectrometry at Arizona: Geochronology of the Climatic Record and Connections with the Ocean

**DOI:** 10.1100/tsw.2002.349

**Published:** 2002-06-11

**Authors:** J.T. Jull, G.S. Burr, J.W. Beck, D.J. Donahue, D. Biddulph, A. L. Hatheway, T. E. Lange, L. R. McHargue

**Affiliations:** NSF Arizona AMS Laboratory, University of Arizona, 1118 E. Fourth Street, Tucson, AZ 85721, USA

**Keywords:** accelerator mass spectrometry, carbon-14, paleoclimate, radiocarbon dating, beryllium-10, corals, forest fires, ENSO

## Abstract

There are many diverse uses of accelerator mass spectrometry (AMS). C studies at our laboratory include much research related to paleoclimate, with C as a tracer of past changes in environmental conditions as observed in corals, marine sediments, and many terrestrial records. Terrestrial records can also show the influence of oceanic oscillations, whether they are short term, such as ENSO (El Niño/Southern Oscillation), or on the millennial time scale. In tracer applications, we have developed the use of I as well as C as tracers for nuclear pollution studies around radioactive waste dump sites, in collaboration with IAEA. We discuss some applications carried out in Tucson, AZ, for several of these fields and hope to give some idea of the breadth of these studies.

## INTRODUCTION

In this paper, we describe our new accelerator mass spectrometry (AMS) system and also summarize some aspects of AMS applications at the University of Arizona facility. Over the last 2 decades, AMS has gradually evolved into the standard method of radionuclide measurements, especially for radiocarbon. At the University of Arizona, we now operate two AMS machines. The first machine is a 2.5-MV Tandetron (General Ionex Corporation), in operation since 1981, and the second, which was installed in spring 2000, is a 3-MV Pelletron (NEC, National Electrostatics Corporation). Most samples run in our laboratory are for radiocarbon measurements, though we have developed applications using $^{10}Be$[1] and $^{129}I$[2]. We have measured over 46,000 radiocarbon samples for a wide variety of applications over the last 20 years at our laboratory.

## AMS RADIOCARBON MEASUREMENTS

### Pretreatment

It is important to consider the careful pretreatment of samples for radiocarbon dating, since improper cleaning can affect the results. Although basic procedures are uniform, pretreatment can vary between different radiocarbon laboratories. The processing of an AMS radiocarbon sample at Arizona follows several well-defined steps, which are summarized below for different types of samples:

1. Organic materials such as charcoal, wood, cellulose, plant material, and animal tissue: after physical inspection, the samples are cleaned with 1 N HCl acid, 0.1% NaOH, and 1 N HCl (acid-base-acid [ABA] pretreatment), washed with distilled water, dried, and combusted at 900°C with CuO.
2. Carbonates: samples are etched with 100% $H_3PO_4$ to remove 50 to 85% of the carbonate, dried, and hydrolyzed with $H_3PO_4$. An alternative is to use selective etching[3].
3. Marine and lacustrine sediments: after cleansing in 1 N HCl and drying, the sample is combusted at 400°C in ~0.3 atm oxygen gas. We will discuss specific problems of sediment dating later in this paper.
4. Textiles, parchment, canvas, art works, and artifacts: the samples are given the ABA pretreatment and, after washing and drying, they are Soxhlet extracted with hexane, then ethanol, and finally methanol. After washing in distilled water and drying, they are combusted at 900°C with CuO[4].
5. Ivory: samples are given a light acid pretreatment or a light ABA pretreatment. Dried samples are combusted at 900°C with CuO.

Once the graphite powder has been produced, the sample is pressed into a target holder of Arizona design, which is now widely used in all NEC ion sources. The measurement of $^{14}C$ follows the procedures and calculations described in detail by Donahue et al.[5,6]

### Improvements to Procedures for Radiocarbon Dating of Sediments

A problem in radiocarbon dating is how to obtain good dates from low-carbon sediments. Many marine sediments have low carbon and obtaining good dates can therefore be a problem. McGeehin et al.[7] discussed separation of different components and low-temperature combustion as a means of addressing this question. The stepped-combustion method of Delqué Kolic[8] was modified at the Arizona AMS laboratory to date pottery samples[9], and we have now applied this procedure to analyze bulk sediments. Each sediment sample is given an ABA pretreatment consisting of 1 M HCl (2 h, 60°C), 0.1 M NaOH (overnight, 60°C), and 1 M HCl (2 h, 60°C). Samples are placed in 9-mm Vycor combustion vessels and are combusted in 0.3 atm ultrapure $O_2$ at 400°C. The $CO_2$ produced at this step is referred to as the “low temperature humin” (LT humin) fraction. After isolating the low temperature $CO_2$, the remaining sample material is then pumped under a high vacuum, recharged with 0.3 atm ultrapure $O_2$ and heated to 900°C. The $CO_2$ produced at 900°C is referred to as the

"high temperature humin" (HT humin) fraction. Both the LT and HT humin $CO_2$ fractions are individually passed through a platinum trap at 1,000°C to oxidize CO, a Copper Oxide/Silver trap to remove volatile gases, and two dry ice/alcohol traps at –78°C to remove water.

## Calculation of Errors

The precision obtained for radiocarbon measurements depends not only on the total counts accumulated in a measurement, but also on the random errors that may occur from fluctuations in parameters of the components of the instrument. That component of the instrument whose small random fluctuations are most likely to produce errors in the measurements is the ion source.

The precision reported with all of our results includes the contribution from an error that we call the "random instrument error" (rme). We have recently made some modifications that reduced this error to ±0.25 from 0.30%. Accuracy is maintained by constantly monitoring the ratio of two standards, oxalic acid Ox-II (made in 1974) and oxalic acid Ox-I (made in 1955), obtained from the U.S. National Institute of Standards and Technology (NIST). Four samples of Ox-II and Ox-I are included in each 32-sample wheel, along with 24 other samples. This ratio, which should be 1.291 for our measurements (1,2), can be used over a long period of time to determine any systematic errors. Systematic errors, deduced from the analysis of these standards measurements, are less than 0.1%. In cases where the Ox-II/Ox-I ratio exceeds our limits, the sample wheel is re-rerun. In very rare cases where this is not possible, an additional error due to this deviation is added to that data.

We also make an additional correction, and error can arise when the samples are smaller than 0.5 mg C. In this case, we make an additional correction. This function was derived from studies of the isotopic ratio measured for small samples of standards, where a decline in the measured ratio is observed as an inverse function of size. The effects are similar to those observed earlier by various AMS laboratories. An additional error ($s_4$) is added quadratically to the previous errors[5,10].

Thus, for modern samples we can measure $^{14}C$ content with precisions of 0.3% on the 3-MV Pelletron and about 0.4% using the Tandetron. If we make three to four independent measurements on identical graphite targets, we can obtain a statistical precision of 0.20 to 0.25%.

## A New 3-MV AMS System

Two years ago, we installed a new 3-MV AMS system based on the NEC 9SDH machine. The low-energy injector includes an NEC MC-SNICS ion source and a 90° magnetic sector. The high-energy end of the machine consists of a 15° electrostatic analyzer, a 90° magnet with a mass-energy product of 176 MeV, and a 77° electrostatic analyzer. A switching magnet has been installed to allow us to switch between different detector systems. The design of the system is indicated in Fig. 1. In Fig. 2, we show the high-energy portion of this machine. The new equipment has improved our measurements of $^{10}Be$ and $^{129}I$, and will allow measurements to be made on the isotopes $^{26}Al$. The higher energy improves the signal-to-noise ratio and thus $^{10}Be$ can be more easily separated from the interfering isobar $^{10}B$. In addition, with the new accelerator, we consistently produce higher Be beam-currents over that of the old accelerator. The NEC machine operates in conjunction with our existing Ionex machine in allowing us to maximize the throughput of $^{14}C$ samples, and currently ~70% of the NEC machine time is dedicated to $^{14}C$.

## Some Examples of AMS Radiocarbon Measurements

### *Extension of the Radiocarbon Time Scale to ~45,000 Years BP*

The calibration of the radiocarbon time scale was originally done by comparing the raw "radiocarbon age" with the known age of tree rings. The current version of the calibration, encompassing tree-ring data back to 11,857 calibrated years before 1950 A.D. and sparse coral data back to ~24,000 years, was published in 1998 by Stuiver et al.[11]. Other records such as speleothems were proposed by Vogel[12] and Vogel and Kronfeld[13]. In the past few years, there have been studies using speleothems[14,15,16], aragonite deposits from the Dead Sea region[17], and attempts to cross-correlate marine foraminifera from Icelandic sea sediments with ice cores[18]. Beck et al.[14] have reported on a possible expansion of the radiocarbon calibration using a new record found in stalagmites from the Bahamas. Many of these records show broad agreement during the period covered by the tree-ring chronology and large fluctuations in $^{14}C$ during the period of 25 to 45 ka. This then also disagrees with the long varved-lake sediment chronology of Kitagawa and van der Plicht[19], which does not show the same fluctuations. A summary of these recent studies in the time period beyond the tree-ring calibration is given in Fig. 2. Beck et al.[14] discussed a record obtained from a stalagmite recovered from an underwater cave in the Bahamas, which was flooded by the last sea-level rise about 11 ka ago. This record provides a nearly continuous record of atmospheric $^{14}C$ from 45 to 11 ka. This record was derived using U, Th, and Pa measurements (obtained by thermal-ionization mass spectrometry [TIMS]) and AMS $^{14}C$ ages. These results reveal elevated and very variable $^{14}C$ between 45 and 33 ka BP. These fluctuations may correlate with peaks in cosmogenic $^{36}Cl$ and $^{10}Be$ isotopes[20], which have been observed in polar ice cores, as well as those observed for $^{10}Be$ in marine sediments[1]. Modeling results suggest that the major features of this record cannot be produced with solar or terrestrial magnetic field modulation alone, but may require significant changes in the carbon cycle [c.f. 14]).

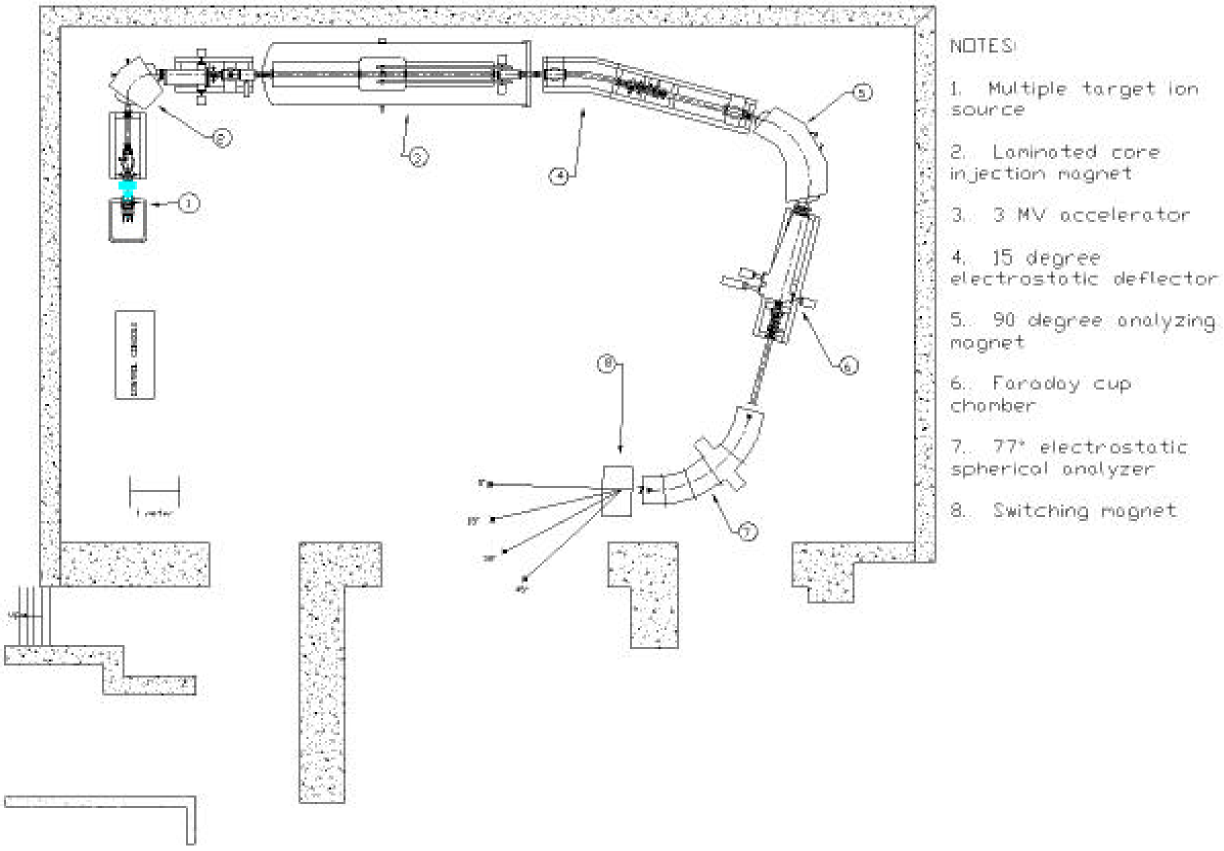

**FIGURE 1.** Layout of the new 3-MV Arizona AMS system, based on a 9SDH Pelletron accelerator.

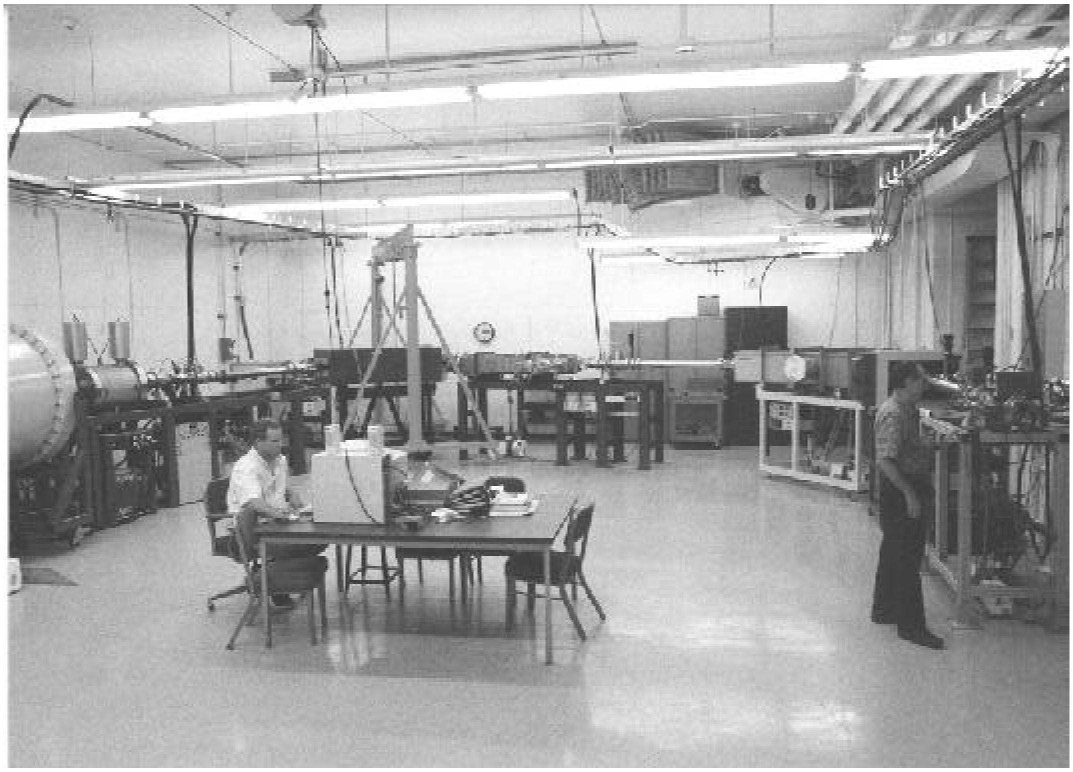

**FIGURE 2.** The high-energy section of the 3MV Pelletron AMS at the University of Arizona. Part of the accelerator is shown on the left, and an electrostatic deflector, the analyzing magnet is also shown on the left. On the right, the 77º electrostatic analyzer and detector system are shown next to Prof. D. Donahue. Mr. D. Biddulph is shown on the computer in the center of the picture.

## *Correlations of Climatic Data in Chinese Loess Deposits*

Loess and paleosol deposits provide an excellent proxy record of climatic change in East Asia[21,22] and in the Americas[23,24]. We have developed a good collaboration with colleagues at the Institute of Earth Environment, Xi'an, to date sequences of loess, paleosol, and other deposits that make up the vast loess deposits in China. The location of these regions in central China is very sensitive to movement of the monsoonal belts. This work has demonstrated that one can show that the fluctuations during the younger Dryas include warm and wet periods, in contrast to the general view of the younger Dryas as a "cold" phenomenon[25,26]. This shows that climatic oscillations may have a very different expression in eastern Asia than the "cold, dry" North Atlantic younger Dryas event[25]. These effects in China appear to be controlled by changes to the flow of monsoonal precipitation[21,22,27]. There is increasing evidence that these local effects may also occur in many different parts of the world. For example, in South America, there appears to be a counterpart to the Younger Dryas "cold" event[28], whereas there is a warm event in the tropical Pacific[29] and a drought in southwestern North America has been discussed[30].

In central Chinese records, the period from 13,000 to 11,000 $^{14}$C years BP (calibrated age 14,900–12,900 years BP) can be identified with the Bølling/Allerød interstadial. During this time, monsoon precipitation clearly increased in the arid to semi-arid zone of central China, a transitional zone between the warm, wet oceanic air from the south and west and the dry winds coming from central Asia. From around 11,200 to 10,000 $^{14}$C years BP (cal 12,900 to 11,600 years BP), which is the Younger Dryas cold period, we find that in Chinese records, this period is characterized by frequent climatic fluctuations. During the mid-Younger Dryas, 10,500 to 10,300 $^{14}$C years BP, several proxy indices[25,31,21,22] showed oscillatory behavior. Similar behavior has been observed in the late

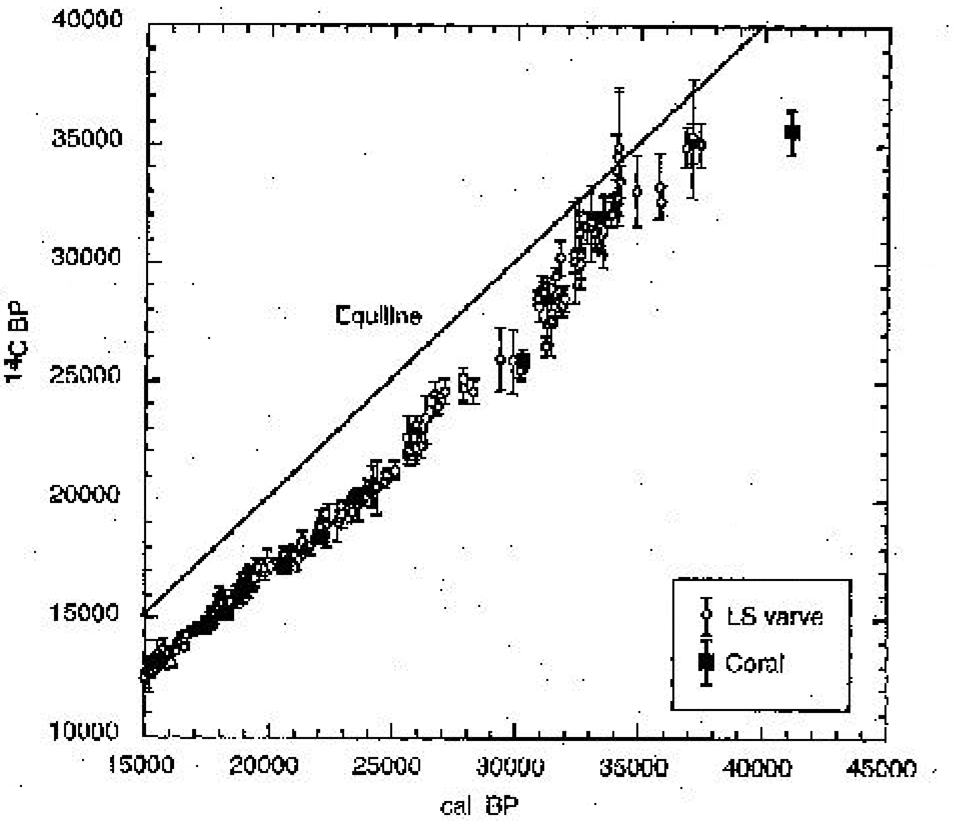

**FIGURE 3.** Extension of the radiocarbon calibration curve compiled from recent publications by Kitagawa and van der Plicht[19] and Beck et al.[14] These results show extensions to the calibration from U-Th in corals and speleothems, as well as lacustrine sediments.

Holocene (5,000 years BP to the present)[32] from Qinghai Lake. Hence, regional climatic changes may be as important as those of a more global nature.

The Holocene record in loess also may show apparent fluctuations on time scales of 2,200 to 2,000 years. These frequencies are often associated with solar effects, as we will discuss later.

## Correlations with Archaeology

Since radiocarbon dates can now be calibrated through the last glacial transition, this allows us to correlate climatic changes with significant events in archaeology and anthropology. This is a result of improved calibrations using tree rings, corals, and also marine varved sediments, aragonite deposits in lake sediments, and speleothems. These all provide new possible calibration methods. This means we can now compare the previously relative radiocarbon dates of a given cultural strata with climatic information from other records, such as the climatic record in ice cores, and we can more clearly define the exact timing of certain archaeological events with climatic change phenomena. One of the most marked climatic events since the end of the full glacial is the Younger Dryas event, which is marked in records in many parts of the world, as we have already mentioned. This cold event was not only rapid in its onset, perhaps less than 100 years, but had a profound effect on climate in the North Atlantic region. It may be expressed in different ways in different parts of the globe and, as just discussed in Asia, oscillatory behavior is observed during this time.

The arrival of early man in the western hemisphere is usually thought to have occurred close to the end of the last Glacial period, since conventional models assume a Bering land bridge route was used. Since the Bering Strait is only 30 m deep at its shallowest, the last sea-level rise would have closed off this route between Asia and the Americas. The Bering land bridge should have remained intact until ~10,000 radiocarbon years BP (11,000 calendar years), when the last significant sea-level rise occurred. Sea-level rise appears to have occurred in two stages of ~60 and ~50 m, as shown by Fairbanks[33] and Edwards et al.[34]

In the southwestern U.S., there has always been a good stratigraphic relationship between the end of the Clovis culture at about 10,900 $^{14}C$ years BP and subsequent evidence for a wetter climate, expressed by algal mat deposits, which dates during the period of the Younger Dryas cold event. Radiocarbon ages on these algal mats also fall into the period of the younger Dryas A cold event at 10,300 to 10,600 years BP[35]. As already mentioned, the possibility of oscillatory climatic behavior during this time has been noted in Asian chronologies[25]. In the European chronology, Amman and Lotter[36] place the Alleröd-younger Dryas boundary in European samples at 10,900" 140 $^{14}C$ years BP, which is close to the conventional definition of ~11,000 $^{14}C$ years BP. The calibrated age of this boundary is 11,490 cal years BP[37,38], close to the 11,600 cal year definition of Alley et al.[39], based on counting annual layers in GISP ice cores, and the estimate of Hughen et al.[40], based on the Cariaco Basin marine sediments.

We believe this is good evidence that the Clovis expansion occurred during the European Alleröd warm period, and that we can likely associate the Clovis drought of Haynes[41] with the Intra-Alleröd cold period (IACP), which occurred about 11,400 to 11,100 $^{14}C$ years BP and was followed by a short warmer epoch before the rapid onset of the Younger Dryas at 10,900 $^{14}C$ years BP. We can also compare these radiocarbon ages to the estimates of sea level rise determined by Bard et al.[42] in Barbados coral and Edwards et al.[34] in the South Pacific. In either case, the algal mat deposits which postdate Clovis, and all Clovis radiocarbon measurements[41,43,44], fall between these two sea-level rise events, as shown in Fig. 4. The start of the Clovis expansion is a little less clear, as the dating of the Monte Verde site in Chile (~12,500 $^{14}C$ years BP)[45] extends the time period for this colonization a little further into the past than earlier estimates.

Another important aspect to the Clovis expansion is not only its apparent climatic connection, but also the connection to the extinction of many species of land mammals. We have already asserted that the Clovis expansion in North America occurred in the Allerod. Almost half of the large land mammals of North America disappeared at the close of the Pleistocene[46]. The Murray Springs site, as discussed above, is only one of many which shows the interplay of climate, the expansion of humans, and the disappearance of large mammals such as mammoths. In a recent paper, Alroy[47] proposed a computer simulation which predicted that extinction would occur between 801 to 1,640 years after the initial arrival of humans. Certainly, this would not contradict existing chronologies of human settlement in the Americas. A related scenario was proposed by Martin[46], who claimed that human effects on the mammal population caused these extinctions, either by hunting or just by the additional environmental pressure placed on these species by the arrival of humans. Hence, very detailed and high-precision chronology is needed to resolve the exact timing of the climatic events, human expansion, and mammoth decline. We also know that megafaunal extinctions resulting from the effects of human expansion appear to have occurred in many other parts of the world, such as Australia[48], New Zealand, and Madagascar, as well as the Americas.

## *Forest-Fire Frequency and Relationship to Climatic Changes*

Another potential proxy for climatic events, with a possible connection to the ocean, is the frequency of forest fires. Molloy et al.[49], Hopkins et al.[50], Horn and Sanford[51], and Carcaillet[52,53] have all used radiocarbon dating to estimate the age of fire events. These studies were performed in very different regions of the world. We have studied the recurrence of forest fires in Yellowstone National Park (U.S.) and showed an interesting periodicity of the order of ~1,000 to 1,500 years[54,55], as shown in Fig. 5. It is interesting to speculate if this is related to the periodic-

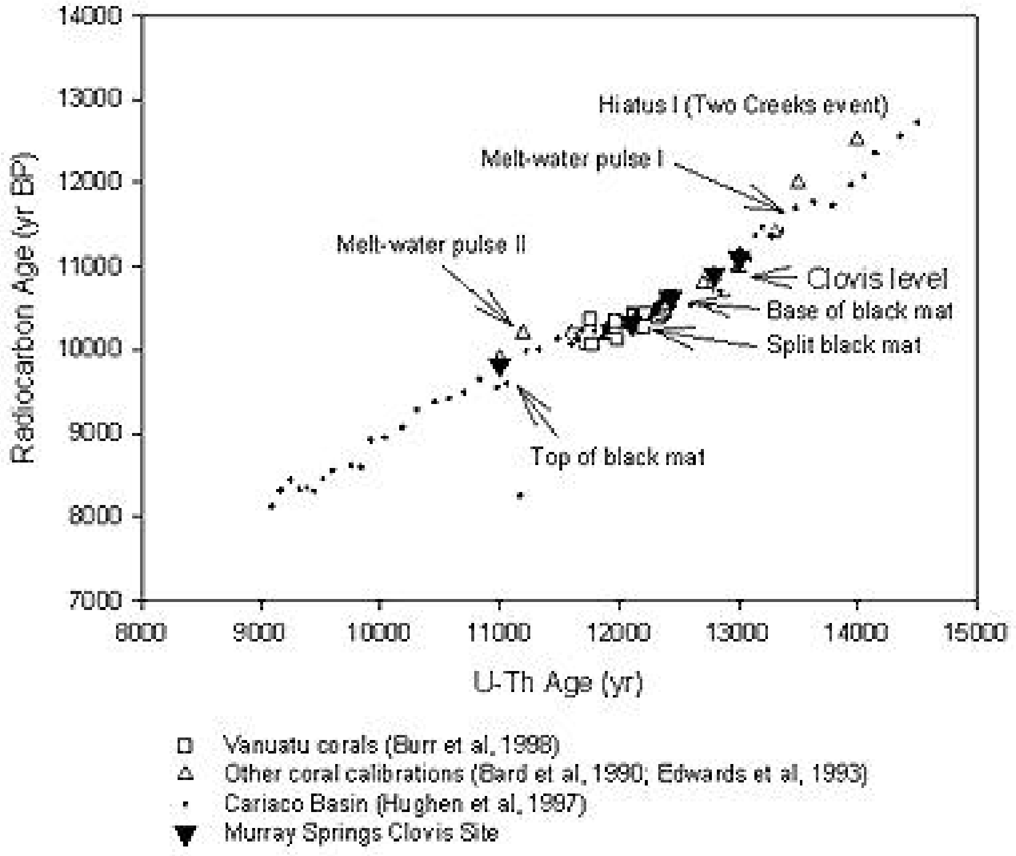

**FIGURE 4.** Relative dating of the expansion of Clovis man in the New World, compared to climatic events during the Late Glacial period. The expansion of early man in the New World is compared to climatic events, especially the sea-level rise determined by Edwards et al.[34] from corals in New Guinea.

ity of the ice-rafting events of Bond et al.[56], but the connection between these two disparate phenomena must be established. Bond et al.[56] argue that this periodicity is associated with solar cycles. Damon and Peristykh[57] have noted solar cycles of similar periodicities in the radiocarbon record. Other studies[58,59] have also suggested a millennium-scale periodicity to fire recurrence in the coastal Pacific Northwest forests. Before one can postulate how climatic change might affect the frequency of forest fires, there are several cautionary notes. First, forest fires may incorporate burning of already dead trees (the "in-built age" of the wood which was burnt). According to Gavin[60], fires may be set deliberately by humans and so the record may be more complex that it at first appears.

## *Modern Carbon Cycle Studies and Studies of El Niño/Southern Oscillation in the Pacific*

Radiocarbon measurements of corals have been used for some time to reveal information about past levels of $^{14}C$ in the surface-mixed layer of the ocean. High frequency analysis of living corals shows that the ocean-mixed layer undergoes considerable temporal and spatial variability in $\Delta^{14}C$. Such variability is due in part to lateral advection of water masses with different isotopic composition around the surface ocean, but in some regions this variability is also linked to fluctuations in the

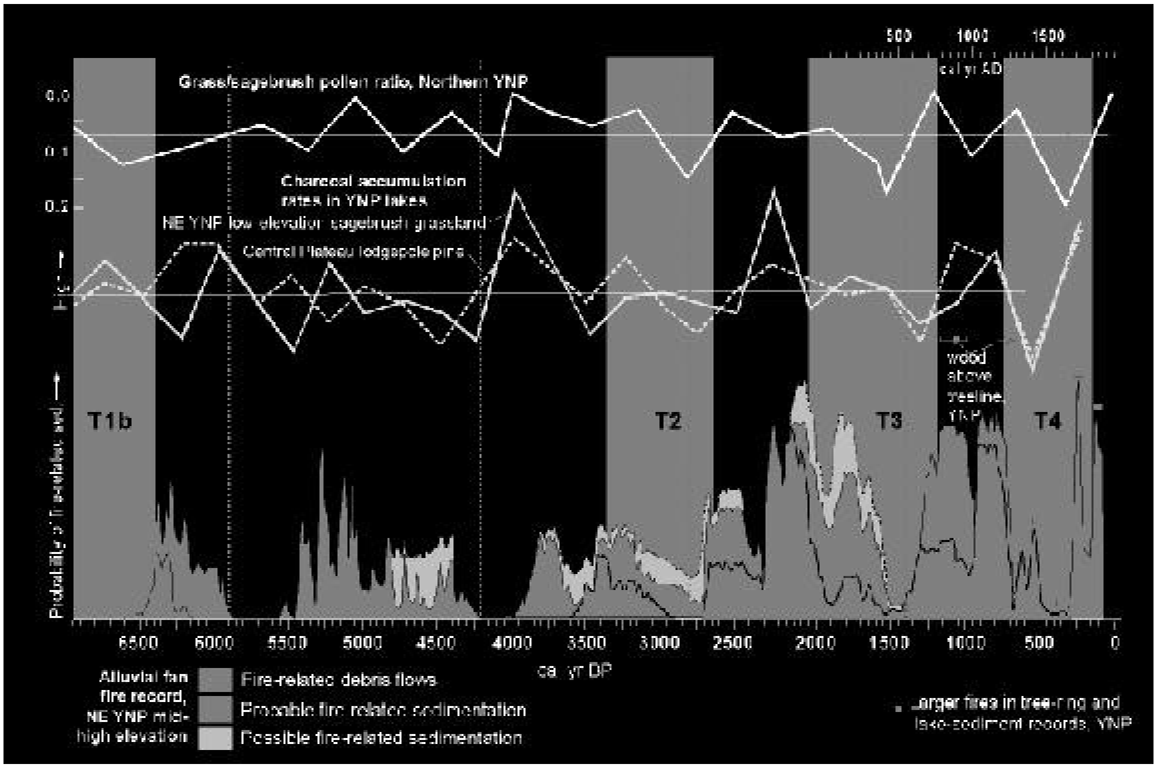

**FIGURE 5.** Probability of forest fires and debris-flow events following fires at Yellowstone National Park, U.S. This plot was derived from radiocarbon dating of charcoal in debris flows and soils[54,55]. (Courtesy of G. Meyer, University of New Mexico.)

upwelling intensity of radiocarbon-depleted waters from the thermocline. Both these phenomena are strongly linked to the El Niño/Southern Oscillation (ENSO). We have studied high frequency $^{14}C$ analysis of corals from several Pacific Ocean regions sensitive to ENSO variability[29]. Thus, these records can be used as a proxy to recreate ENSO frequency and intensity over the past several centuries. Equally important is the observation that ENSO exerts a strong control on the level of $CO_2$ emissions from the tropical Pacific, which is the principal oceanic source region for $CO_2$ emissions to the atmosphere. One example of these kinds of effects on $^{14}C$ is the correlation of sea-surface temperature records with $\Delta^{14}C$ in Hawaiian corals discussed by Druffel et al.[61] (see Fig. 6).

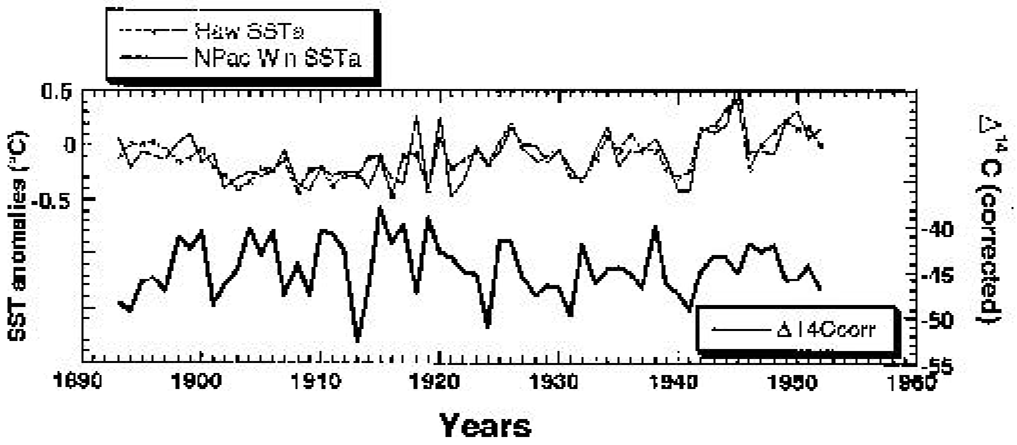

**FIGURE 6.** Winter sea-surface temperature anomalies in the North Pacific (30 to 50°N, 180–120°W), Hawaiian mean annual SST, and annual $\Delta^{14}C$ record for corals from Hawaii, showing a positive correlation between SST and $\Delta^{14}C$ (adapted from Druffel et al.[61]).

Here, ENSO-like cycles affect regional oceanic circulation. Since mixed layer $\Delta^{14}C$ is in part a monitor of thermocline/mixed layer $CO_2$ flux, which in turn modulates ocean/atmosphere $CO_2$ fluxes, these coral measurements give us an opportunity to study the pattern of variations for this important carbon cycle flux term. Our work has largely focused on analysis of corals from Christmas Island in the equatorial Pacific. However, there are also preliminary records from Vanuatu, The Marqueses, and Easter Island in the South Pacific Ocean, all regions that are representative of major water masses or are near major current boundaries.

## Some AMS $^{10}Be$ Measurements

We now make all our $^{10}Be$ measurements to the 3-MV NEC machine described above. Sample preparation involves dissolution of the sample and addition of 1-mg Be carrier, extraction of Be using acetylacetone, and ion exchange chromotography, as discussed by McHargue et al.[1] $Be(OH)_2$ is precipitated from the final solutions and is converted to BeO by heating in air. The BeO powder is then mixed with a silver powder as a binder and the mixture is pressed into a Cu target holder for the AMS measurements.

## Correlations Between $^{14}C$ and $^{10}Be$ Chronology

We can obtain independent evidence from $^{10}Be$ data in marine sediments of these large fluctuations in cosmic-ray production rates in the period of 30 to 40 ka[1,62]. The production of $^{10}Be$ is directly related to the intensity of the cosmic-ray flux on the Earth's upper atmosphere, which in turn is a function of the primary cosmic-ray flux, and modulation by the heliomagnetic field and geomagnetic field[63,64].

$^{10}Be$, once formed by spallation of O and N in the atmosphere by cosmic rays, is quickly removed from the atmosphere by precipitation. The intensity of the geomagnetic dipole-field of the Earth can thus be inferred from the concentrations of $^{10}Be$ sequestered over time in marine sediments. There are, however, other effects. McHargue et al.[65] studied $^{10}Be$ in a partly varved marine core from the Gulf of California (Leg 64, DSDP 480) and they confirmed a prominent $^{10}Be$ anomaly (see Fig. 7) first noted in Antarctic ice by Raisbeck et al.[66] This "spike" may be correlated with the excursions in $\Delta^{14}C$ we have observed in speleothem records. Subsequently, McHargue et al.[62] also determined that $^{10}Be$ measured from a core (CH88-10P) from the Blake Outer Ridge correlated remarkably well with the paleointensity of the geomagnetic field independently measured from the same core. The variance of the concentration of $^{10}Be$ in the authigenic fraction of the sediments closely correlated with the inverse of the variance in the paleointensity of the geomagnetic field, but can lag up to 1,000 years of sedimentation. By contrast, the data from several other elements, some climatically sensitive, and from beryllium show no relationship to $^{10}Be$ nor to the paleomagnetic data. This lack of correlation indicates these other elements are deposited independent of the magnetic field, unlike $^{10}Be$.

Superimposed on these large-scale trends in $^{14}C$ and $^{10}Be$ are millennial and submillennial variations in many records (e.g., tree rings, ice cores) that may coincide with abrupt shifts in climate. These signals can be observed in the tree ring and coral records: Edwards et al.[34] observed a large increase in $\Delta^{14}C$ at the start of the Younger Dryas. More recently, Lange et al.[67] discuss large (70 per mil) fluctuations in $\Delta^{14}C$ at the time of another hiatus (~12,200 radiocarbon year BP) in the $^{14}C$ record coincident with the "Older Dryas" cold event. This approximately marks a cold period after the start of the Bolling period. Since the record of $CO_2$ in the atmosphere, derived from ice cores, indicates that $CO_2$ increased gradually[68], the change of $CO_2$ in the atmosphere alone cannot be the cause of these variations. It would appear some combination of change in production rate and ocean circulation is responsible [c.f. 14,34]. We are attempting to improve these records in several reservoirs: tree rings, stalagmites, and corals. We will discuss some other possible proxy records that show such fluctuations later in this paper.

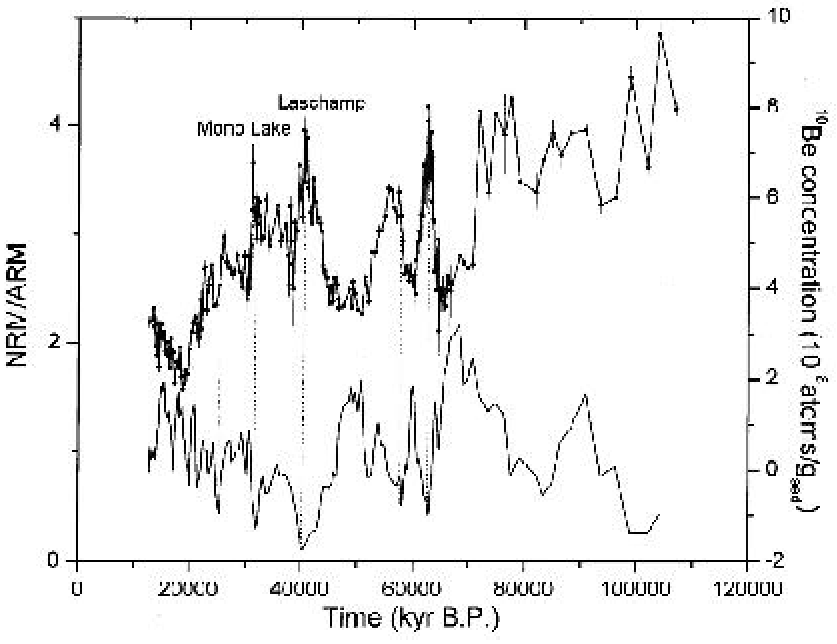

**FIGURE 7.** $^{10}$Be concentrations (top line) from marine sediments from the Outer Blake Ridge off the southeastern coast of the U.S. (water depth ~3,800 m). NRM/ARM (Natural Remanent Magnetization normalized to Anhysteretic Magnetization) was measured from the same sediment samples[71] as those processed for $^{10}$Be and is shown in the lower curve. Increases in $^{10}$Be correlate with decreases in the paleomagnetic dipole field and are shown to be consistent with theoretical models for the relationship between the production of $^{10}$Be in the atmosphere and the intensity of the geomagnetic field[62]. Sediments older than 65 ka are carbonate rich and thus interpretations of the intensity of the paleomagnetic signal are considered to be less reliable. The Laschamp geomagnetic excursion is interpreted from the work on this core and others on the Blake Ridge by Schwartz et al.[71] The Mono Lake excursion was assigned by correlation of $^{10}$Be between the Blake Ridge and sediments from the Gulf of California[65].

## CONCLUSIONS

AMS allows the measurement of small samples for $^{14}$C, $^{10}$Be, $^{129}$I, and other radionuclides. It is the key method for determination of these nuclides. Its use for radiocarbon dating has displaced the former methods of counting radioactive decay, due to orders of magnitude smaller sample requirements. For longer-lived nuclides such as $^{10}$Be, AMS has made routine measurements possible, where radioactive decay counting was very difficult. We have only been able to present a few of the many applications studied in our laboratory in this paper. Besides the applications discussed, which focus on natural levels of these radionuclides and their relation to paleoclimate, AMS is also useful for studying tracers in the ocean. Isotopic tracers such as $^{14}$C and $^{129}$I allow us to follow ocean circulation and also anthropogenic pollution in the oceans (e.g., Jull et al.[69] and Povinec et al.[70]).

## ACKNOWLEDGMENTS

We thank the staff of the NSF Arizona AMS Laboratory for their technical support. The work reported in this paper was funded in part by U.S. National Science Foundation grants EAR 97-30699, EAR98-71035 and EAR01-15488.

## ADDITIONAL REFERENCES

Alley, R.B., Meese, D.A., Shuman, C.A., Gow, A.J., Taylor, K.C., Grootes, P.M., White, W.C., Ram, M., Waddington, E.D., Mayewski, P.A., and Zielinski, G.A. (1993) Abrupt increase in Greenland snow accumulation at the end of the Younger Dryas. *Nature* **362**, 527–529.

Amman, B.S. and Lotter, A.F. (1987) Late-glacial radiocarbon and palynostratigraphy on the Swiss plateaus. *Boreas* **18**, 109–126.

Burr, G.S., Beck, J.W., Taylor, F.W., Récy, J., Edwards, R.L., Cabioch, G., Corrège, T., Donahue, D.J., and O'Malley, J.M. (1998) High resolution radiocarbon calibration between 11.7 and 12.4 kyr BP derived from $^{230}$Th ages of corals from Espiritu Santo Island, Vanuatu. *Radiocarbon* **40**, 1085–1092.

Burr, G.S., Thomas, J.M., Reines, D., Jeffrey, D., Courtney, C., Jull, A.J.T., and Lange, T. (2001) Sample preparation of dissolved organic carbon in groundwater for AMS analysis. Proc. 17$^{th}$ Int. Radiocarbon Conference, Judean Hills, Israel. *Radiocarbon* **43**, 183–190.

Haynes, Jr., C.V. (1987) Curry Draw, Cochise County, Arizona: a late Quaternary stratigraphic record of Pleistocene extinction and Paleo-Indian activities. In *Geological Society of America Centennial Field Guide, Cordilleran Section.* Geological Society of America, Boulder, CO. pp.23–28.

Hodell, D.A., Brenner, M., Curtis, J.H., and Guilderson, T. (2001) Solar forcing of drought frequency in the Maya lowlands. *Science* 292, 1367–1370.

Lal, D., and Jull, A.J.T. (2001) In-situ cosmogenic 14C: production and examples of its unique applications in studies of terrestrial and extraterrestrial processes. *Radiocarbon* **43**, 731–742.

Stuiver, M. and Polach, H. (1977) Discussion: reporting of 14C data. *Radiocarbon* **19**, 355–363.

Stuiver, M., Reimer, P.J., Bard, E., Beck, J.W., Burr, G.S., Hughen, K.A., Kromer, B., McCormac, G., van der Plicht, J., and Spurk, M. (2000) INTCAL98 Radiocarbon age calibration 24,000-0 cal. BP. *Radiocarbon* **40**, 1041–1083.

Tuniz, C., Bird, J.R., Fink, D., and Herzog, G.F. (1998) *Accelerator Mass Spectrometry: Ultrasensitive Analysis for Global Science*. CRC Press, Boca Raton, FL. 371pp.

van der Plicht, J. (2000) The 2000 radiocarbon varve/comparison issue. *Radiocarbon* **42**, 313–322

Yokoyama, Y., Esat, T.M., Lambeck, K., and Fifield, L.K. (2000) Last ice age millenial scale climatic changes recorded in Huon peninsula corals. *Radiocarbon* **42**, 383–401.
